# Triphala Churna—A Traditional Formulation in Ayurveda Mitigates Diabetic Neuropathy in Rats

**DOI:** 10.3389/fphar.2021.662000

**Published:** 2021-06-03

**Authors:** Sachin V. Suryavanshi, Kalyani Barve, Veeranjaneyulu Addepalli, Sachin V. Utpat, Yogesh A. Kulkarni

**Affiliations:** ^1^ Shobhaben Pratapbhai Patel School of Pharmacy & Technology Management, SVKM’s NMIMS, Mumbai, India; ^2^ MES Ayurveda Mahavidyalaya, Ghanekhunt-Lote, Ratnagiri, India

**Keywords:** diabetic neuropathy, oxidative stress, NGF, streptozotocin, triphala

## Abstract

Neuropathy is a common complication of diabetes affecting a large number of people worldwide. Triphala churna is a formulation mentioned in Ayurveda-a traditional system of medicine. It is a simple powder formulation consisting of powders of three fruits, *Emblica officinalis* L.*, Terminalia bellirica* (Gaertn.) Roxb*.* and *Terminalia chebula* Retz. Individual components of Triphala churna have anti-diabetic and antioxidant activities. Hence, this study was designed to evaluate the effect of Triphala churna on diabetic neuropathy. Diabetes was induced with streptozotocin (STZ, 55 mg/kg, *i. p.*) in rats. Animals were grouped and treated orally with Triphala churna at a dose of 250, 500, and 1,000 mg/kg after 6 weeks of diabetes induction for the next 4 weeks. At the end of study, parameters such as body weight, plasma glucose level, motor nerve conduction velocity were determined. The effect of Triphala churna on thermal hyperalgesia, mechanical hyperalgesia, and mechanical allodynia was also determined at the end of study. The plasma cytokine levels like TGF-β1, TNF-α, and IL-1β were determined by ELISA assay. Histopathology study of the sciatic nerve was studied. Western blotting was performed to study the expression of neuronal growth factor.Treatment with Triphala churna showed a significant reduction in plasma glucose and a significant rise in body weight. Triphala treatment significantly increased the motor nerve conduction velocity and decreased the thermal and mechanical hyperalgesia, as well as mechanical allodynia. The treatment significantly inhibited levels of circulatory cytokines like TGF-β1, TNF-α, and IL-1β. Histopathology study confirmed the neuroprotective effect of Triphala churna. The expression of NGF was significantly increased in sciatic nerves after treatment with Triphala churna. From the results, it can be concluded that Triphala churna delays the progression of neuropathy in diabetic rats.

## Introduction

Uncontrolled chronic hyperglycemic condition can cause damaging effects on organs such as heart, kidney, eye, and nerve leading to vascular complications. Diabetic neuropathy (DN) is one of the common microvascular complications affecting the majority diabetic population worldwide. DN is characterized by degenerative neuronal loss due to alterations in nerve damage and repair process which result in progressive loss of sensation (Charnogursky et al., 2014). Diabetic neuropathy can be broadly classified into autonomic neuropathy and peripheral neuropathy (Aslam et al., 2014). The prevalence of diabetic peripheral neuropathy (DPN) ranges from 16 to 87% with painful diabetes-related neuropathy about 26%. The lower limb amputation is 10–20-fold more common in diabetic patients as compared to non-diabetic patients (IDF, 2019). The characteristics of DPN include loss of sensory and motor nerves. Chronic hyperglycemia contributes to pathological changes like demyelination of nerves, narrowing of neuronal capillary, axonal thickening, neuronal damage, and loss of nerve fibers (Sasaki et al., 2020). The neuronal damage can be attributed to elevated levels of oxidative stress and advanced glycation end products (AGEs) in nerves (Suryavanshi and Kulkarni, 2017).

In recent years, various natural products have been evaluated in the management of diabetic neuropathy. Triphala churna is a powder formulation in Ayurveda, a well-known traditional system of medicine. This formulation has been reported to have potent antioxidant activity, immunomodulatory, anti-inflammatory, hypoglycemic activities (Peterson et al., 2017). It has also shown potent antiglycation activity (Ganeshpurkar et al., 2015). The individual components of Triphala churna viz. *Terminalia bellirica* (Gaertn.) Roxb., *Terminalia chebula* Retz., and *Emblica officinalis* L. have been reported to have an anti-diabetic potential and potent anti-oxidant property (Ansari et al., 2014; D’souza et al., 2014; Nampoothiri et al., 2011; Sabu and Kuttan, 2009). Recently, Triphala churna has been reported for its beneficial effects in diabetic nephropathy (Suryavanshi et al., 2020). Gallic acid and ellagic acid are the hydrolyzable tannins present in Triphala churna have reported for their beneficial effect on diabetic nephropathy (Garud and Kulkarni, 2018; Zhou et al., 2019). Gallic acid has shown a neuroprotective effect via reduction in inflammation and oxidative stress (Diaz et al., 2021). Triphala churna may show neuroprotective effect via inhibiting inflammation, oxidative stress, and by reducing neuronal damage. Hence, the present study was designed to study the effect of Triphala churna in diabetic neuropathy in rats.

## Materials and Methods

### Chemicals

2-thiobarbituric acid, 1,1,3,3-tetramethoxypropane, nitrobluetetrazolium, streptozotocin (STZ), and reduced glutathione were procured from Sigma Aldrich (United States).

### Collection and Authentication of Plant Material

The fruits of *Terminalia bellirica* (Gaertn.) Roxb., *Terminalia chebula* Retz., and *Emblica officinalis* L. were procured from D.G. Ayurvedic Sangraha, Mumbai, India. All fruit samples were authenticated from Agharkar Research Institute, Pune, Govt. of India, India. Standardization of these fruits was carried out by using high-performance thin-layer chromatography (HPTLC) by using gallic acid and ellagic acid as marker compounds (Supplementary material).

### Preparation of Triphala Churna

Fruits of *Terminalia chebula* Retz., *Terminalia bellirica* (Gaertn.) Roxb., and *Emblica officinalis* L. were powdered. The powders were passed through sieve No 80. The Triphala churna was prepared by mixing an equal proportion of powdered fruits (Ayurvedic Formulary of India, 2003).

### Experimental Animals

Male *Sprague Dawley* (SD) rats (180–220 g) were procured from the National Institute of Biosciences, Pune, India. The animals were acclimatized to standard laboratory conditions (Temperature 22 ± 2°C, Humidity 75 ± 5%, 12 h light, and 12 h dark cycle) for one week and then maintained throughout the study. Animals had free access to a basal nutritional diet and purified water *ad libitum*. The necessary approvals for animal experimentation were taken from the Institutional Animal Ethics Committee (Approval No: CPCSEA/IAEC/P-63/2016) which was formed as per guidelines of the Committee for the Purpose of Control and Supervision of Experiments on Animals (CPCSEA), Govt. of India. NIH guidelines were followed while handling and experimentation on animals (NIH, 2011).

### Induction of Diabetes and Treatment

Diabetes induction was done with a single dose administration of STZ (55 mg/kg, *i. p.*) which was dissolved in ice-cold citrate buffer (0.1 M, pH- 4.4) (Kulkarni and Garud, 2016). Induction of diabetes was confirmed by measuring plasma glucose level after 48 h of STZ administration. Animals with glucose levels above 250 mg/dl were included in the study. Animals were randomized into different groups after six weeks of induction according to their glucose level and body weight. One group of diabetic animals was kept as diabetic control which received vehicle [0.5% w/v carboxymethyl cellulose (CMC) solution]. Other groups of diabetic animals received Triphala churna at a dose of 250, 500, and 1,000 mg/kg. One group of non-diabetic animals was kept as normal control which received CMC solution. Treatments were given orally once a day for 28 days.

## Evaluation Parameters

### Body Weight and Plasma Glucose Level

The body weight was recorded at the end of study. The blood was collected from retro-orbital plexus in tubes containing disodium salt of ethylenediaminetetraacetic acid (EDTA) as an anticoagulant. Plasma was separated through cold centrifugation (2,500 rpm for 15 min at 4°C) and was stored at −20°C until further use. Plasma glucose level was determined on a biochemical analyzer (Erba Mannheim, Germany) using commercially available kits (Transasia Biomedicals Ltd. India).

### Hot Plate Test

The thermal hyperalgesia was assessed using Eddy’s hot plate test. At the end of study, animals were subjected to a hot plate (IITC Life Science, United States) which was maintained at 55 ± 0.5°C. Response latency was recorded as the time required for the first response (Licking, Jumping, or Flickering of hind paw). The cut-off time was set to 15 s to avoid tissue damage (Kulkarni et al., 2015).

### Tail Immersion Test

At the end of study, effect of Triphala churna on the nociceptive threshold to thermal stimuli in diabetic rats was determined using a tail immersion test. The tail of an animal was immersed in hot water which was maintained at 55 ± 0.5°C. The response latency was considered as the time taken by the animal to remove the tail from hot water. The cut-off time was set to 15 s to avoid tissue damage (Padgaonkar et al., 2018).

### Mechanical Hyperalgesia

At the end of study, effect of Triphala churna treatment on mechanical hyperalgesia was evaluated using the Randall-Selitto test. After completion of treatment, rats were restrained in slings. A constantly increasing pressure stimulus was applied to the dorsal surface of the hind paw of the rat with a pressure applicator (IITC Life Science, United States). The response pressure (maximum pressure at which rats showed first response—either paw withdrawal, struggling, or squeaking) was recorded using a digital meter (IITC Life Science, United States). The cut-off pressure was set to 400 g to avoid tissue damage. Triplicate readings were recorded for each animal separated by at least 20 min interval and mean values of triplicate reading were taken as final readings (Al-Rejaie et al., 2015).

### Mechanical Allodynia

The Von Frey test was carried out to determine the effect of Triphala churna on a nociceptive threshold (mechanical allodynia) to stimuli. After 4-weeks treatment, rats were kept in a cage with a mesh bottom. Animals were allowed to acclimatize with a cage for 20 min. After acclimatization, A constantly increasing pressure stimulus was applied Von Frey pressure applicator attached with rigid tip (IITC Life Science, United States) to the ventral surface of hind paw of a rat. The maximum response pressure (pressure at which rat showed paw withdrawal or brisk lifting) was recorded with a digital meter (IITC Life Science, United States). The cut-off pressure was set to 400 g to avoid tissue damage. Triplicate readings were recorded for each rat separated by at least 20 min interval and mean values of triplicate reading were taken as final readings (Romanovsky et al., 2010).

### Motor Nerve Conduction Velocity

Motor nerve conduction velocity (MNCV) was determined after completion of treatment in the sciatic posterior tibial conducting system using a data acquisition system (Iworx, United States) as previously described (Sayyed et al., 2006; Bhatt and Addepalli, 2010). Rats were anesthetized with urethane (1.2 g/kg, i. p.). The sciatic nerve was stimulated by applying an 8 V single stimulus using bipolar needles (24 gauge) placed proximal to the sciatic notch. The receiving electrodes were placed in the tibial muscles (proximal to the ankle). The M wave reflexes were recorded digitally using a data acquisition system (Iworx, United States). Distance between stimulation point and receiver point was recorded manually. Time required to show M wave reflex was considered as conduction time. MNCV was calculated by conduction time divided by the distance between the stimulating and recording electrode.

### Oxidative Stress Parameters

After completion of treatment, animals were humanely sacrificed. The sciatic nerves were separated from muscles and carefully isolated. Hundred mg of tissue was mixed with 0.5 ml of phosphate buffer (0.1 M, pH 7.4) and homogenized by using a probe homogenizer (Polytron PT 2500E, Kinematica, Switzerland). The tubes were placed in ice to avoid temperature rise. The protein content of the homogenate was measured according to the previously described method (Lowry et al., 1951).

Different fractions of the tissue homogenates were prepared and further processed to obtain post-nuclear and post mitochondrial fractions. The whole homogenate was used for Malondialdehyde (MDA) (Ohkawa et al., 1979) and reduced glutathione (GSH) assay (Ellman, 1959). One fraction was subjected to centrifugation at 2,500 rpm for 20 min at 4°C to extract post-nuclear supernatant (PNS). This supernatant was used to determine catalase activity (Luck, 1965). Superoxide dismutase (SOD) assay was carried out with post mitochondrial supernatant (PMS) (Paoletti et al., 1986). The PMS was obtained by centrifuging homogenate at 10,000 rpm for 20 min at 4°C.

### ELISA Assay

The interleukin—1β (IL—1β), tumor necrosis factor—α (TNF—α), and transforming growth factor-β1 (TGF-β1) present in the plasma were estimated using ELISA kits as per the manufacturer’s instructions (RayBiotech, United States).

### Western Blotting

Western blotting of sciatic nerve tissue was carried out as per standard protocol (Randolph et al., 2007). Tissues were homogenized with probe homogenizer (Polytron PT 2500E, Kinematica, Switzerland) in ice-cold radioimmunoprecipitation assay (RIPA) lysis buffer (5 ml/g tissue) containing protease inhibitors cocktail (Thermo Fisher Scientific, United States). Homogenate was further centrifuged at 4°C with 10,000 rpm for 15 min and the supernatant was used to estimate protein concentration. Total protein was estimated by the Lowry method (Lowry et al., 1951). Protein samples were loaded with Laemmli sample buffer (1X) and separated on 10% sodium dodecyl sulfate-polyacrylamide gel electrophoresis (SDS–PAGE) using electrophoresis and blotting apparatus (Bio-Rad, United States). Separated proteins were transferred onto PDVF membrane (0.45 μm, Millipore, United States) and blocked for 1 h with 3% w/v Bovine serum albumin at room temperature. The membrane was further incubated overnight at 4°C with an anti-NGF antibody and anti-β-actin antibody (Santa Cruz Biotechnology, United States) at dilution 1:1,000. After adequate washing, the membrane was incubated with a secondary antibody (m-IgGκ BP-HRP, Santa Cruz Biotechnology, United States) for 1 h at dilution 1:1,250 at room temperature. Protein expression was visualized using an enhanced chemiluminescence method and X-ray film. Photographs of X-ray film were taken using a photographic scanner. Band intensity was determined as measure of NGF expression using software Image Studio™ Lite Version 5.2 (LI-COR Biosciences, United States).

### Histopathology

The sciatic nerves were detached from surrounding muscles and carefully isolated. The nerves were further fixed in neutral formalin buffer and then embedded in paraffin wax. Thin sections of 5 μm were taken with a microtome (Leica Biosystems, Germany). The sections were then deparaffinized, rehydrated, and stained with Mayer’s hematoxylin and eosin (HE) stain. The slides were observed for the lesions, leukocytic infiltration, fragmentation of myelin and axon, neuronal swelling and degeneration, chromatolysis, inflammation, neural edema, and proliferation of Schwann cells and glial cells. The histopathological evaluation was carried out by a qualified histopathologist who was blind to treatment groups. The severity of the lesions was scored as no abnormality detected 0), minimal changes 1), mild changes 2), moderate changes 3), and distribution was recorded as focal multifocal and diffuse 4).

### Statistical Analysis

All the data were analyzed by one-way ANOVA followed by a *post-hoc* Tukey test. Data were analyzed using graph pad prism software (version 5). *p* < 0.05 were considered to be significant.

## Results

### Effect of Triphala Churna Treatment on Body Weight and Plasma Glucose Levels

**FIGURE 1:**
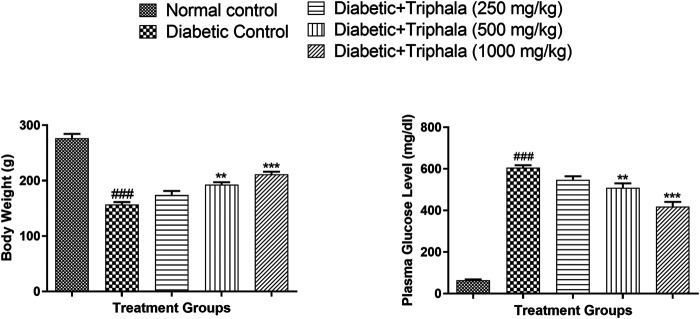
Effect of Triphala churna on body weight and plasma glucose level. Data are expressed as mean ± S.E.M. (*n* = 6), ^###^ *p* < 0.001 when compared to normal control, **p < 0.001, ***p < 0.001 when compared to diabetic control.

Diabetic animals showed a significant decrease in body weight (*p* < 0.001) when compared to animals in the normal group. Treatment with Triphala churna showed significant improvement in body weight of the diabetic animals at the dose of 500 mg/kg (*p* < 0.01) and 1,000 mg/kg (*p* < 0.001) when compared with the diabetic control group [F (4, 25) = 57.88]. Diabetic animals showed a significant rise in plasma glucose when compared to animals in the normal control group. Treatment with Triphala churna significantly decreased plasma glucose level at a dose of 500 mg/kg (*p* < 0.01) and 1,000 mg/kg (*p* < 0.001) when compared to animals in the diabetic control group [F (4, 25) = 162.4] (Figure 1).

### Effect of Triphala Churna Treatment on Behavioral Parameters

Rats in the diabetic control group showed a significant reduction in response latency in the hot plate test (*p* < 0.001) when compared with animals in the normal control group. In hot plate test, the response time was significantly increased in Triphala treated animals at a dose of 250 mg/kg (*p* < 0.01), 500 mg/kg (*p* < 0.001), and 1,000 mg/kg (*p* < 0.001) when compared to animals in the diabetic control group [F (4, 25) = 103.0]. In tail immersion test, Triphala treatment significantly increased the response latency at a dose of 500 mg/kg (*p* < 0.05) and 1,000 mg/kg (*p* < 0.001) when compared to animals in the diabetic control group [F (4, 25) = 32.39].

**FIGURE 2:**
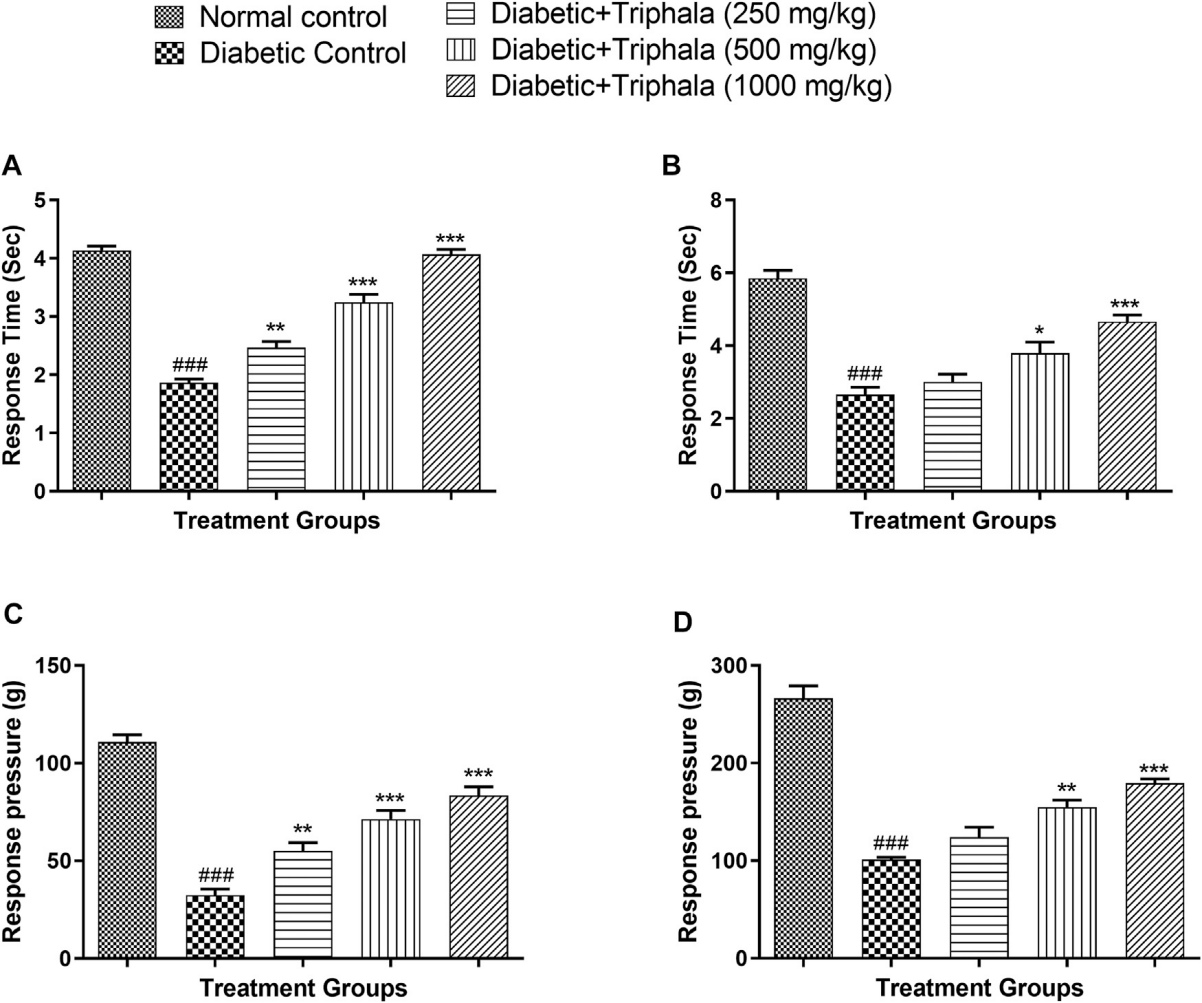
Effect of Triphala churna in **(A)** Hot plate test; **(B)** Tail immersion test; **(C)** Von Frey test; **(D)** Randall Selitto test. Data are expressed as mean ± S.E.M. (*n* = 6), ^###^ *p* < 0.001 when compared to normal control, *p < 0.05, **p < 0.001, ***p < 0.001 when compared to diabetic control.

In the Von Frey test, the diabetic animals showed a significant decrease in response pressure which was significantly increased in Triphala treated animals at a dose of 250 mg/kg (*p* < 0.01), 500 mg/kg (*p* < 0.001), and 1,000 mg/kg (*p* < 0.001) when compared to diabetic control [F (4, 25) = 54.34]. In the Randall Selitto test, animals in the diabetic control group showed a significant decrease in response pressure (*p* < 0.001) when compared to normal control animals which were significantly increased in Triphala treated animals at a dose of 500 mg/kg (*p* < 0.01) and 1,000 mg/kg (*p* < 0.001) when compared to diabetic control animals [F (4, 25) = 58.33] (Figure 2).

### Effect of Triphala Churna Treatment on Motor Nerve Conduction Velocity (MNCV)

**FIGURE 3:**
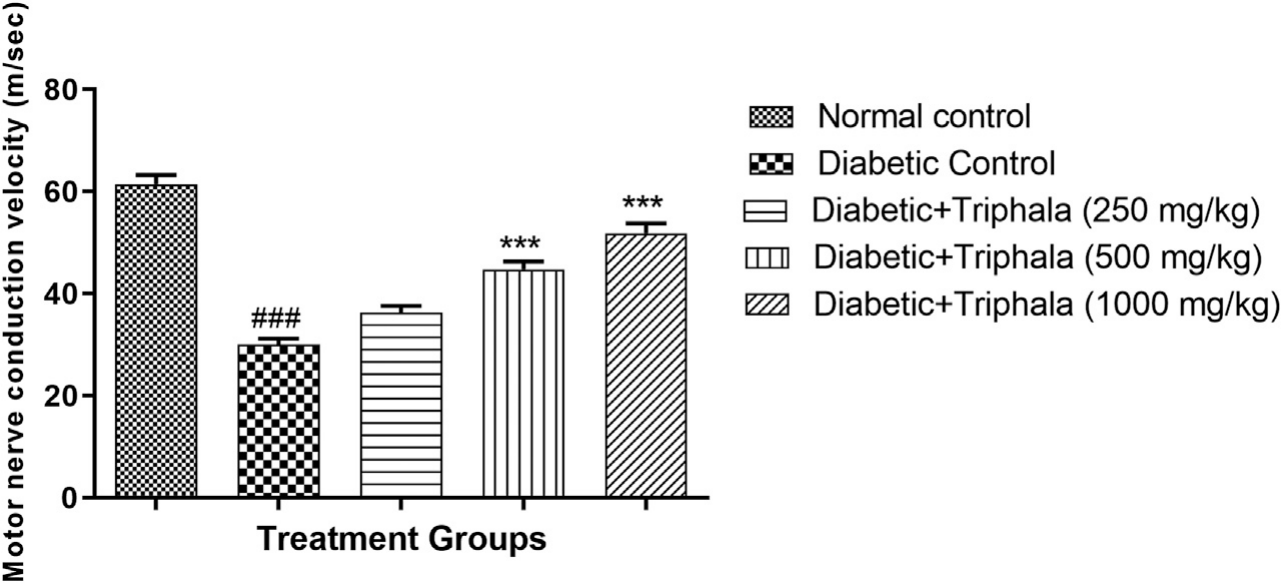
Effect of Triphala churna on motor nerve conduction velocity. All data is expressed as Mean ± SEM (*n* = 6), ^###^ *p* < 0.001 when compared with normal control, ***p < 0.001 when compared with diabetic control.

Animals in the diabetic control group showed significant reduction (*p* < 0.001) in motor nerve conduction velocity when compared with animals in the normal control group. Treatment with Triphala churna showed a significant increase in MNCV at a dose of 500 and 1,000 mg/kg [F (4, 25) = 60.13, *p* < 0.001] when compared to animals in the diabetic control group. (Figure 3).

### Effect of Triphala Churna on Oxidative Stress Parameters

Animals in the diabetic control group showed a significant increase in MDA levels and a significant decrease in GSH, Catalase and SOD levels when compared to animals in the normal control group. The MDA levels were decreased significantly in Triphala churna treated animals at all selected dose levels when compared to the diabetic control group. Treatment with Triphala churna also significantly increased the levels of GSH, catalase, and SOD at a dose of 500 and 1,000 mg/kg when compared to animals in the diabetic control group (Table 1).

**TABLE 1:** Effect of Triphala churna on oxidative stress parameters.

Parameter group	MDA (nmol/mg protein)	GSH (μmole/mg protein)	Catalase (µmole of H_2_O_2_ decomposed/min/mg protein)	SOD (unit/mg protein)
Normal control	2.50 ± 0.21	37.77 ± 2.24	0.128 ± 0.006	0.91 ± 0.07
Diabetic control	13.14 ± 1.00^###^	22.11 ± 1.64^###^	0.055 ± 0.004^###^	0.38 ± 0.05^###^
Diabetic + triphala (250 mg/kg)	7.43 ± 0.46^***^	26.82 ± 2.95	0.072 ± 0.004	0.57 ± 0.02
Diabetic + triphala (500 mg/kg)	5.34 ± 0.24^***^	31.56 ± 1.16^*^	0.080 ± 0.006^*^	0.62 ± 0.05^*^
Diabetic + triphala (1,000 mg/kg)	2.91 ± 0.42^***^	34.05 ± 2.12^**^	0.093 ± 0.006^***^	0.86 ± 0.05^***^
F (DFn, DFd)	F (4, 25) = 62.74	F (4, 25) = 8.452	F (4, 25) = 28.22	F (4, 25) = 19.41

All data is expressed as Mean ± SEM (*n* = 6). ^###^ *p* < 0.001 when compared with normal control, *p < 0.05, **p < 0.01, ***p < 0.001 when compared with diabetic control.

### Effect of Triphala Churna on Inflammatory Markers

Diabetic animals showed a significant increase in plasma TGF-β1, TNF-α, and IL-1β levels when compared with normal control animals. Treatment with Triphala churna significantly reduced the plasma TGF-β1 and TNF-α levels at a dose of 500 mg/kg (*p* < 0.05), and 1,000 mg/kg (*p* < 0.001) when compared to diabetic control animals. The IL-1β levels were also significantly decreased in Triphala churna treated animals at a dose of 250 mg/kg (*p* < 0.05), 500 mg/kg (*p* < 0.01), and 1,000 mg/kg (*p* < 0.001) when compared to diabetic control (Table 2).

**TABLE 2:** Effect of Triphala churna on inflammatory markers in diabetic neuropathy.

Parameter Group	TGF-β1 (ng/ml)	TNF-α (pg/ml)	IL-1β (pg/ml)
Normal control	34.1 ± 2.4	482.2 ± 8.0	844.0 ± 21.9
Diabetic control	54.5 ± 1.3^###^	643.3 ± 14.1^###^	1,177.0 ± 42.2^###^
Diabetic + triphala (250 mg/kg)	43.6 ± 4.1	607.2 ± 30.3	1,041.0 ± 28.9*
Diabetic + triphala (500 mg/kg)	39.7 ± 2.0*	562.8 ± 18.1*	990.7 ± 37.8**
Diabetic + triphala (1,000 mg/kg)	34.9 ± 3.8***	521.1 ± 15.2***	910.7 ± 22.3***
F (DFn, DFd)	F (4, 25) = 8.102	F (4, 25) = 11.99	F (4, 25) = 16.23

All data is expressed as Mean ± SEM (*n* = 6). ^###^ *p* < 0.001 when compared with normal control, *p < 0.05, **p < 0.01, ***p < 0.001 when compared with diabetic control.

### Effect of Triphala Churna on NGF Expression

**FIGURE 4:**
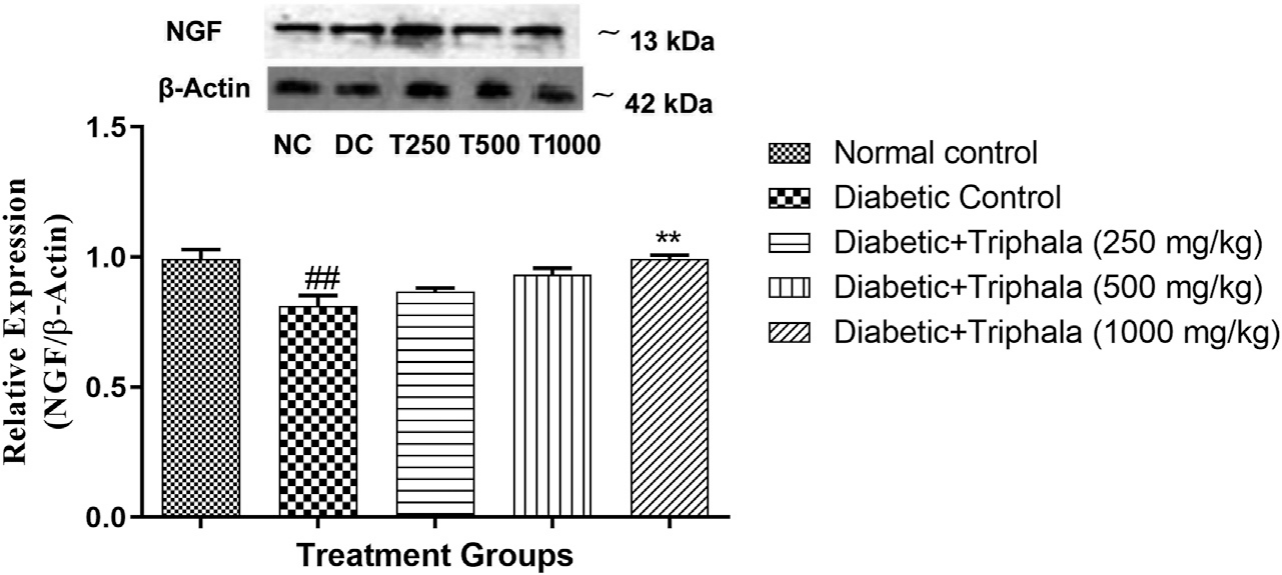
Effect of Triphala churna on NGF expression. All data is expressed as Mean ± SEM (*n* = 3). ###*p* < 0.001 when compared with normal control, **p < 0.01 when compared with diabetic control.

The expression of neuronal growth factor (NGF) was significantly decreased in animals in the diabetic control group as compared to the normal control group. Treatment with Triphala churna significantly increased NGF expression at a dose of 1,000 mg/kg [F (4, 10) = 8.602, *p* < 0.01] when compared to animals in the diabetic control group (Figure 4).

### Histopathology

**FIGURE 5:**
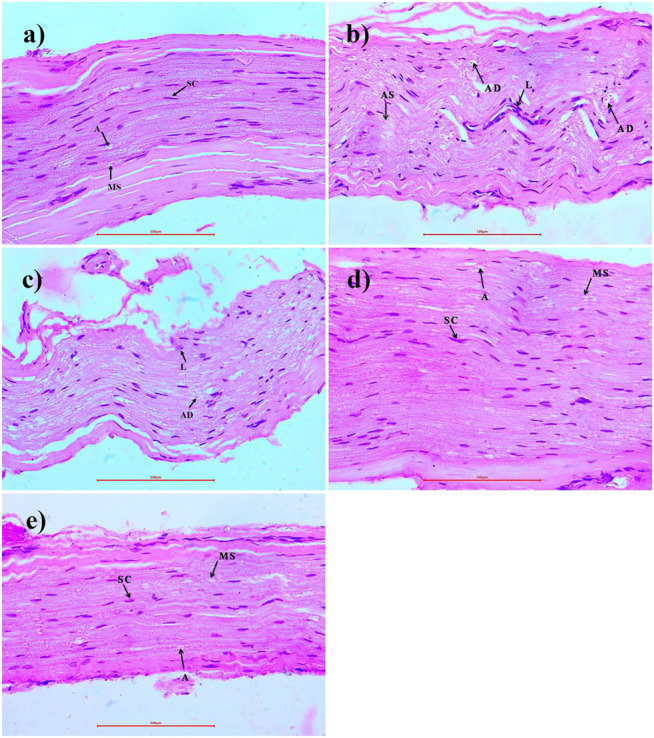
Effect of Triphala churna on sciatic nerve tissue - H.E. staining (400X). **(A)** Normal control: Sciatic Nerve showing normal histology, myelin sheath (MS), Schwann cells (SC), axon (A). **(B)** Diabetic control: showing degeneration of axon (AD), lymphocytic infiltration (L), axonal swelling (AS). **(C)** Diabetic + Triphala (250 mg/kg): showing degeneration of axon (AD), lymphocytic infiltration (L). **(D)** Diabetic + Triphala (500 mg/kg): showing normal histology, myelin sheath (MS), Schwann cells (SC), axon (A). **(E)** Diabetic + Triphala (1,000 mg/kg): showing normal histology, myelin sheath (MS), Schwann cells (SC), axon (A).

Microscopic examination of sciatic nerve of rats of different treated groups showed various lesions viz., focal mild proliferation of schwann cell (Normal control: 0/3, Diabetic control: 1/3, Diabetic + Triphala 250 mg/kg: 0/3, Diabetic + Triphala 500 mg/kg: 0/3, Diabetic + Triphala 1,000 mg/kg: 0/3), focal minimal to mild lymphocytic infiltration (Normal control: 0/3, Diabetic control: 2/3, Diabetic + Triphala 250 mg/kg: 2/3, Diabetic + Triphala 500 mg/kg: 0/3, Diabetic + Triphala 1,000 mg/kg: 0/3), multifocal mild axonal degeneration (Normal control: 0/3, Diabetic control: 2/3, Diabetic + Triphala 250 mg/kg: 2/3, Diabetic + Triphala 500 mg/kg: 0/3, Diabetic + Triphala 1,000 mg/kg: 0/3), focal minimal to mild axonal swelling (Normal control: 0/3, Diabetic control: 2/3, Diabetic + Triphala 250 mg/kg: 1/3, Diabetic + Triphala 500 mg/kg: 0/3, Diabetic + Triphala 1,000 mg/kg: 0/3). Sciatic nerves of diabetic control rats showed mild to moderate neuropathic lesions. Treatment with Triphala churna at 500 mg/kg and 1,000 mg/kg delayed the development of neuropathic lesions in the sciatic nerve (Figure 5).

## Discussion

In diabetic neuropathy, persistent hyperglycemic conditions are responsible for increased autoimmune damage, insufficiency of neurochemical growth factors, and demyelination of autonomic neurons leading to peripheral nerve injury (Addepalli and Suryavanshi, 2018; Vinik et al., 2013). The damage to capillary blood vessels and peripheral nerves leads to the generation of ROS and hypoxic conditions in the highly vascular organs (Boulton et al., 2005; Fang et al., 2004; Pittenger et al., 2003). Activation of the polyol pathway, overburden of oxidative stress, accumulation of glycated hemoglobin, and collagen deposition are considered as major risk factors in diabetic neuropathy leading to nerve injury (Suryavanshi and Kulkarni, 2021).

Triphala churna is a well-known formulation in the traditional system of medicine—Ayurveda. Traditionally, Triphala churna is administered up to 3–6 g daily to treat various ailments in humans (API, 2011). The human equivalent dose for a rat was calculated from the human dose according to body surface area and was considered as the middle dose (FDA, 2005).

Diabetes is a metabolic disorder caused by either insulin insufficiency or insulin resistance. Dysregulation of insulin release or insulin activity leads to depletion of glucose transport and thereby weight loss and increased plasma glucose levels. The weight loss and rise in blood glucose level were reversed by Triphala treatment.

Peripheral neuropathy is associated with increased perception to vibration and thermal stimulus which further progresses to sensory loss due to neuronal damage. The thermal, mechanical hyperalgesia and mechanical allodynia have been reported in considerable diabetic patients (Obrosova, 2009). The allodynia and hyperalgesia conditions can be attributed to various factors such as impaired neurotrophic support, impaired activities of aldose reductase, COX-2, inflammatory cytokines, inhibition of release of neurotransmitters like Gamma-Aminobutyric Acid (GABA), and depletion of spinal potassium-chloride cotransporters (Obrosova, 2009). Triphala has been traditionally used to relieve pain and other ailments (Baliga et al., 2012; Peterson et al., 2017). The Triphala treatment significantly reduced the thermal and mechanical hyperalgesia and mechanical allodynia in diabetic rats.

The polyol pathway plays an important role in diabetic neuropathy. Persistent hyperglycemia increases polyol flux in the autonomic nerves which in turn accumulate sorbitol and fructose (Finegold et al., 1983). Increased levels of sorbitol and fructose decrease the levels of myoinositol and impair Na+/K + ATPase activity in the nerves which cause axonal degeneration and demyelination of nerve fibers (Greene and Lattimer, 1984; Pittenger et al., 2003; Vinik and Ziegler, 2007). Simultaneously, increased oxidative stress in the nerves activates inflammatory mediators. This results in degeneration of neuronal microvasculature *via* endothelial hyperplasia, vessel wall thickening, and capillary closure due to collagen deposition (Ewing and Clarke, 1986). Degeneration of nerves affects the nerve conduction velocities. The Triphala treatment increased the nerve conduction velocity to a significant extent.

Chronic hyperglycemic conditions predominantly increase secretion of cytokines such as TNF-α and IL1-β which in turn causes overexpression of cell adhesion molecules, TGF-β, and other inflammatory markers. These mediators mediate the remodeling or accumulation of extracellular matrix and axonal degeneration (Ristikj-Stomnaroska et al., 2019). Inhibition of inflammatory cytokines can inhibit neuronal degeneration. The Triphala churna has been reported for its anti-inflammatory effect *via* inhibition of TGF-β1, TNF-α, and IL-1β (Kalaiselvan and Rasool, 2016). The inhibition of the inflammatory cascade may provide neuroprotection by reducing leukocytic infiltration, and demyelination of neurons. Hence, in the present study, the effect of Triphala churna on TGF-β1, TNF-α, and IL-1β was evaluated. Triphala treated animals showed a significant reduction in inflammatory cytokines indicating neuroprotection.

Oxidative stress plays a central role in neuronal degeneration by activating inflammatory cascade, extracellular modulation, and increased lipid peroxidation (Bhatt and Addepalli, 2012; Suryavanshi and Kulkarni, 2017). Alteration of antioxidant enzymes such as catalase, glutathione, and superoxide dismutase precipitates mitochondrial dysfunction and demyelination of neurons (Suryavanshi and Kulkarni, 2020). Triphala churna reduced the MDA levels and increased the activity of antioxidant enzymes such as catalase, SOD, and GSH. This effect of Triphala churna may be attributed to its potent antioxidant activity.

Persistent hyperglycemia increased ROS generation, and increased advanced glycation end products cause neuronal damage *via* increasing leukocytic infiltration and reduction in nerve growth factor (NGF). The impaired NGF levels further lead to altered nerve growth, vascular functions, loss of nutritive support to peripheral nerves, and thereby nerve damage (Pittenger et al., 2003). Triphala treatment improved the expression of NGF in peripheral nerves (Sciatic nerve) indicating its protective effect against nerve damage.

Histopathology study supported the findings by confirming the reduction in lymphocytic infiltration, demyelination of sciatic nerve, and axonal swelling. Treatment with Triphala churna inhibited neuronal damage *via* inhibition of inflammatory cascade, TNF-α, IL1-β, and TGF-β inhibition thereby modulation in the extracellular matrix and demyelination of neurons.

Tannins possesss anti-diabetic, anti-inflammatory, anti-glycative insulin sensitizing activites (Laddha and Kulkarni, 2019). Gallic acid and ellagic acid are major phytochemicals belonging to the class of tannins present in the Triphala churna. Reports have confirmed the beneficial role of gallic acid in the management of diabetes and diabetic complications such as diabetic nephropathy and cardiomyopathy, neuropathy due to its potent antioxidant, anti-inflamamtory TGF-β inhibitory activity and by increasing insulin sensitivity (Abdel-Moneim et al., 2017; Garud and Kulkarni, 2018; Patel and Goyal, 2011; Raafat and Samy, 2014). Ellagic acid has also shown beneficial effects in the management of diabetes and its associated complications *via* its anti-oxidant, anti-glycative action and *via* regulation of inflammatory signalling (Ahad et al., 2014; Chao et al., 2010; D’souza et al., 2014; Zhou et al., 2019). Thus the beneficial effects of the Triphala in the diabetic neuropathy may be linked with presence of the tannins mainly gallic acid and ellagic acid.

## Conclusion

From the results, it can be concluded that Triphala churna has beneficial effects in diabetic neuropathy which may be attributed to decreased oxidative stress, inhibition of inflammatory cytokines and increased expression of NGF in rats.

## Data Availability

The raw data supporting the conclusion of this article will be made available by the authors, without undue reservation.
