# Supplementary material for: Triphala Churna—A Traditional Formulation in Ayurveda Mitigates Diabetic Neuropathy in Rats
Source: Front Pharmacol. 2021 Jun 3;12:662000. doi: 10.3389/fphar.2021.662000 (PMC8211421; doi:10.3389/fphar.2021.662000)
Supplement: Supplementary file 1 [file DataSheet1.PDF]

Supplementary Material

Standardization

Dried fruits of *Terminalia chebula, Terminalia bellirica* and *Emblica officinalis* were coarsely powdered and subjected for extraction. The aqueous and alcoholic extracts were prepared by cold maceration technique. 10 g of drug was macerated separately with water and alcohol for 24 h then filtered through Whatman filter (pore size 0.45 µm). The filtrates were dried on water bath.

High Performance Thin Layer Chromatography (HPTLC) of *T. chebula, T. bellirica,* and *E. officinalis* using gallic acid as marker compound.

Gallic acid (Sigma Aldrich, St. Louis, USA) was used as a marker compound. An AS-30 HPTLC sample applicator (Desaga, Germany) was used to apply samples and densitogram were recorded on CD-60 densitogram (Desaga, Germany).

Preparation of standard solutions:

A standard stock solution (1 mg/ml) was prepared by dissolving 10 mg of accurately weighed gallic acid and making up the volume to 10 ml with methanol. The stock solution was further diluted with methanol to give a working solution of gallic acid (50, 100, 150, 200 and 250 µg/ml).

Preparation of test solutions:

Accurately weighed (100 mg) aqueous and alcoholic extracts of fruits of Terminalia chebula, Terminalia belliricaand Amalaki (*Emblica officinalis* Gaertn.) were diluted to 20 ml (5mg/ml) with water and alcohol respectively.

Chromatographic conditions:

Pre-coated silica gel 60 F_{254} TLC aluminium plates (E. Merck Ltd., Mumbai) 0.2 mm thickness were used for estimation. Toluene : Ethyl acetate : Formic acid (5:3.5:0.5) was finalized as solvent system for development of TLC**.** Densitogram was recorded at 254 nm.

Calibration curve:

A specific volume of (10 µl) of the different concentrations of working solutions (50, 100, 150, 200 and 250 µg/ml) were applied on TLC plate as bands with an AS-30 HPTLC sample applicator

(Dessaga, Germany). The TLC was developed by Toluene : Ethyl acetate : Formic acid (5:3.5:0.5) as a solvent system in triplicate through chamber, to distance of 8cm. The TLC was dried and densitogram was recorded at 254 nm. The calibration curve was prepared by plotting area under curve (AUC) *Vs* concentration of gallic acid.

Estimation of gallic acid in the crude drug

A specific volume of (10 µl) of the aqueous and alcoholic extracts was applied on TLC plate as bands with an AS-30 HPTLC sample applicator (Dessaga, Germany). The TLC was developed in solvent system in triplicate through chamber, to distance of 8cm. The TLC was dried and densitogram was recorded at 254 nm. Concentration of gallic acid was calculated from the calibration curve of gallic acid.

HPTLC of *T. chebula, T. bellirica,* and *E. officinalis* using ellagic acid as marker compound

Ellagic acid (Sigma Aldrich, St. Louis, USA) was used as a marker compound. An AS-30 HPTLC sample applicator (Dessaga, Germany) was used to apply samples and densitogram were recorded on CD-60 densitogram (Dessaga, Germany).

Preparation of standard solutions:

A standard stock solution (1 mg/ml) was prepared by dissolving 10 mg of accurately weighed ellagic acid in methanol and making up the volume to 10 ml with methanol. The stock solution was further diluted with methanol to give different working solutions of ellagic acid (20, 40, 60, 80 and 100 µg/ml).

Preparation of test solutions:

Accurately weighed (100 mg) aqueous and alcoholic extracts of fruits of Terminalia chebula, Terminalia belliricaand Amalaki (*Emblica officinalis* Gaertn.) were diluted to 20 ml (5 mg/ml) with water and alcohol respectively.

Chromatographic conditions:

Pre-coated silica gel 60 F_{254} TLC aluminium plates (E. Merck Ltd., Mumbai) 0.2 mm thickness were used for estimation. Toluene : Ethyl acetate : Formic acid (5:3.5:0.5) was finalized as solvent system for development of TLC**.** Densitogram was recorded at 254 nm (Jeganathan and Kannan, 2008; Rajasekaran et al., 2011).

Calibration curve:

A specific volume of (10 µl) of the different concentrations of working solutions (20, 40, 60, 80 and 100 µg/ml) were applied on TLC plate as band with an AS-30 HPTLC sample applicator (Desaga, Germany). The TLC was developed by Toluene : Ethyl acetate : Formic acid (5:3.5:0.5) as a solvent system in triplicate through chamber, to distance of 8cm. The TLC was dried and densitogram was recorded at 254 nm. The calibration curve was prepared by plotting area under curve (AUC) *Vs* concentration of ellagic acid

Estimation of ellagic acid in the crude drug

A specific volume of (10 µl) of the aqueous and alcoholic extracts was applied on TLC plate as bands with an AS-30 HPTLC sample applicator (Dessaga, Germany). The TLC was developed in solvent system in triplicate through chamber, to distance of 8cm. The TLC was dried and densitogram was recorded at 254 nm. Concentration of ellagic acid was calculated from the calibration curve of ellagic acid (Jeganathan and Kannan, 2008; Rajasekaran et al., 2011).

Results

High Performance Thin-Layer Chromatography (HPTLC)

Estimation of gallic acid by HPTLC

Estimation of gallic acid in different extracts was performed using HPTLC. he aqueous extract of *Terminalia chebula* was found to contain 17.50±0.25 %w/w, and alcoholic extract 8.04±0.09 %w/w of gallic acid. Aqueous extract of *Terminalia bellirica* was found to contain 7.95±0.18 %w/w, while alcoholic extract showed 13.34±0.10 %w/w of gallic acid. Aqueous extract of *Emblica officinalis* showed 19.62±0.32 %w/w, while alcoholic extract showed 8.09±0.15%w/w of gallic acid.

Table 1 Estimation of gallic acid by HPTLC

**Name of Extract**	Gallic acid (%w/w)
Aqueous extract of *Terminalia chebula*	17.50±0.25
Aqueous extract of *Terminalia bellirica*	7.95±0.18
Aqueous extract of *Emblica officinalis*	19.62±0.32

Alcoholic extract of *Terminalia chebula*	8.04±0.09
Alcoholic extract of *Terminalia bellirica*	13.34±0.10
Alcoholic extract of *Emblica officinalis*	8.09±0.15

Values were expressed as Mean ± SEM (n=3)

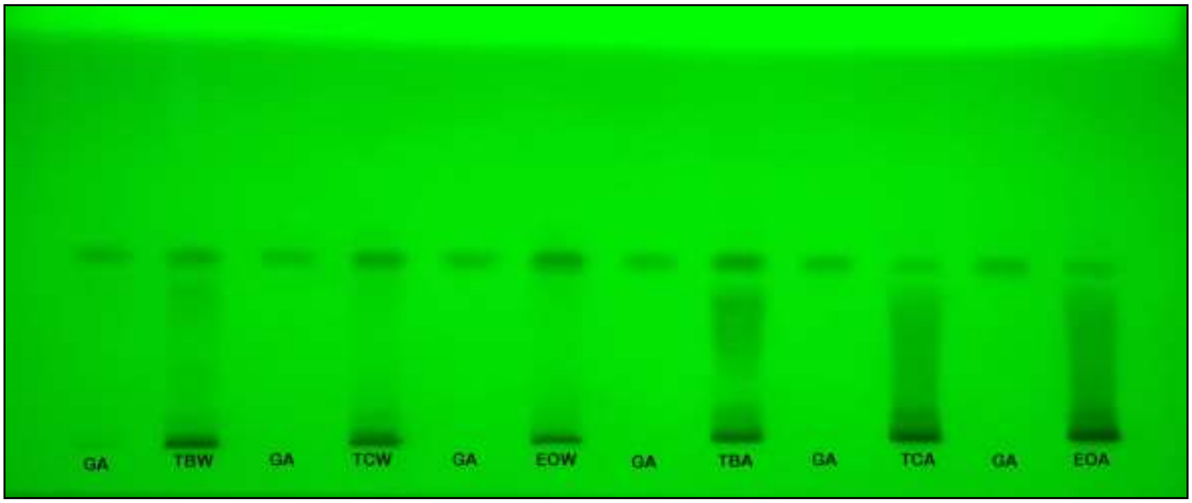

Figure 1 HPTLC of Triphala churna using gallic acid marker at 254nm

GA: Gallic acid, **TBW**: Aqueous extract of *Terminalia bellirica*, **TCW**: Aqueous extract of *Terminalia chebula*, **EOW**: Aqueous extract of *Emblica officinalis*, **TBA**: Alcoholic extract of *Terminalia bellirica*, **TCA**: Alcoholic extract of *Terminalia chebula*, **EOA**: Alcoholic extract of *Emblica officinalis*,

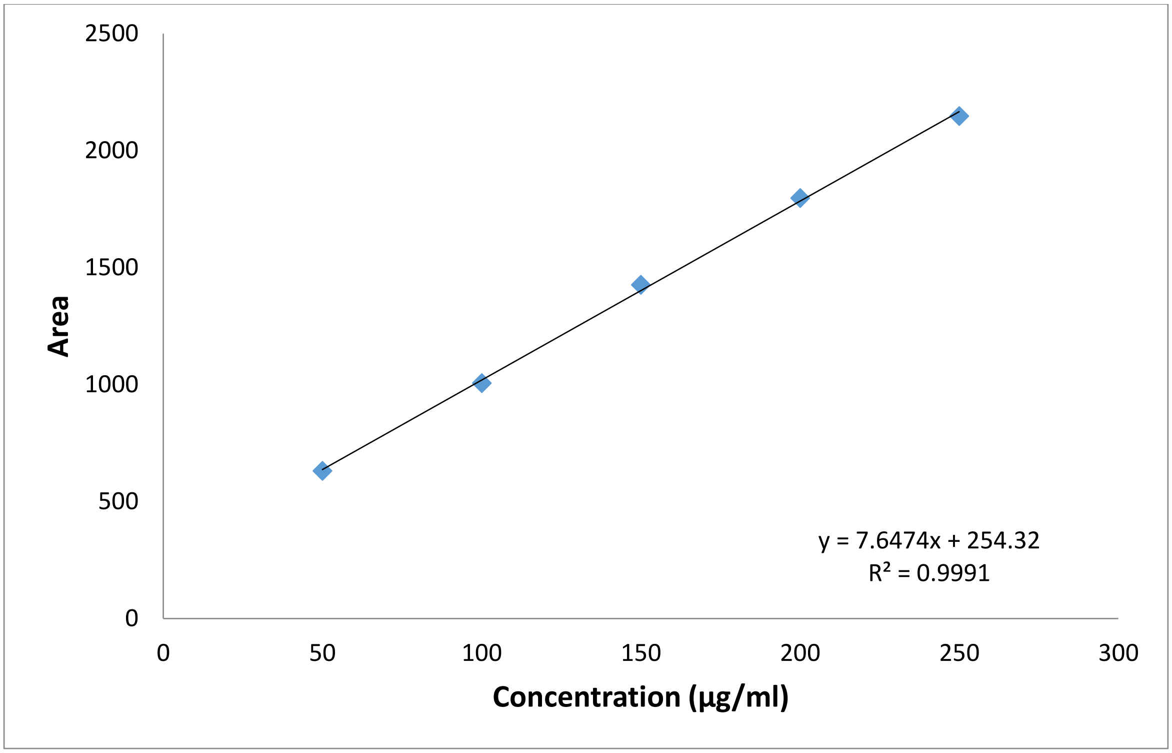

Figure 2 Calibration curve of gallic acid by HPTLC

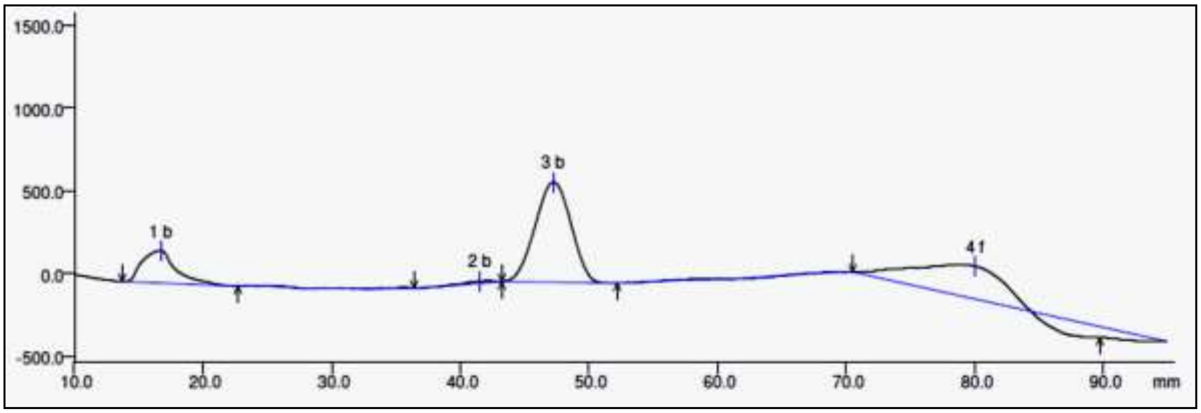

Figure 3 Chromatogram of standard gallic acid by HPTLC

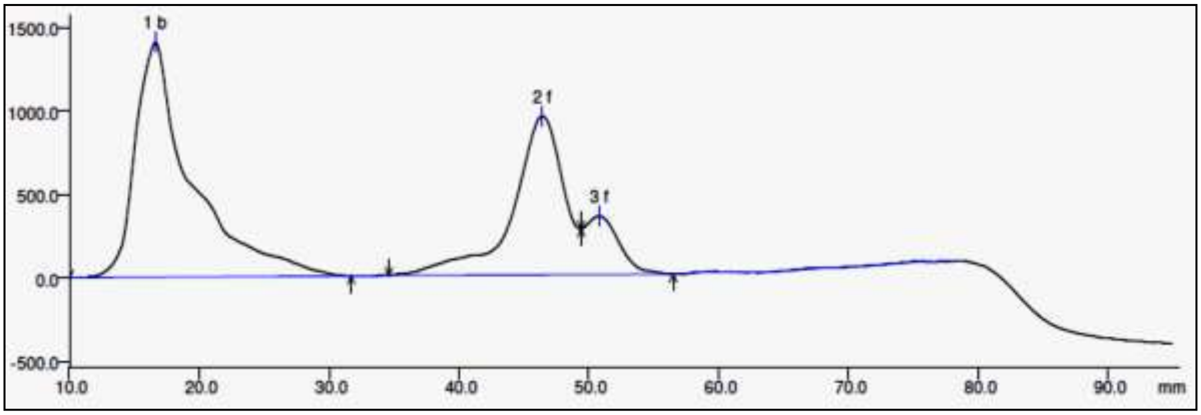

Figure 4 Chromatogram of aqueous extract of Haritaki (*Terminalia chebula*)

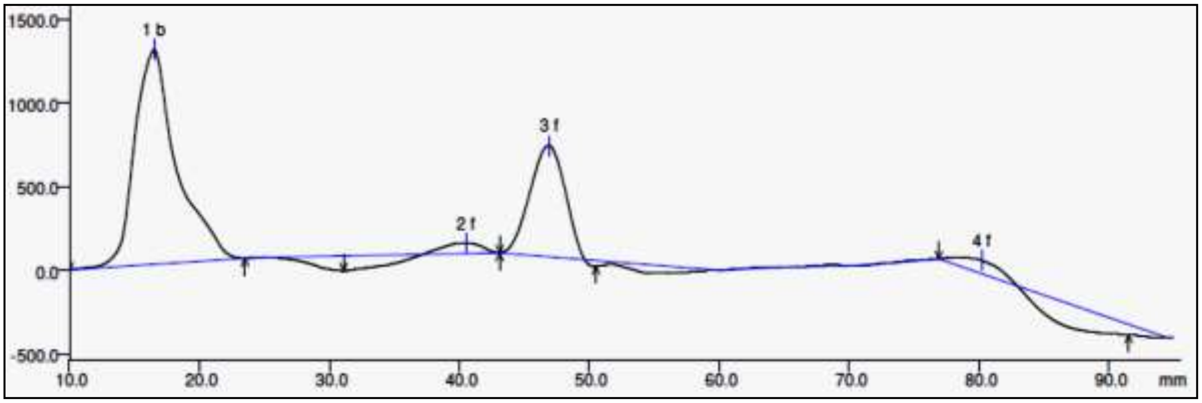

Figure 5 Chromatogram of aqueous extract of Bibhitaki (*Terminalia bellirica*)

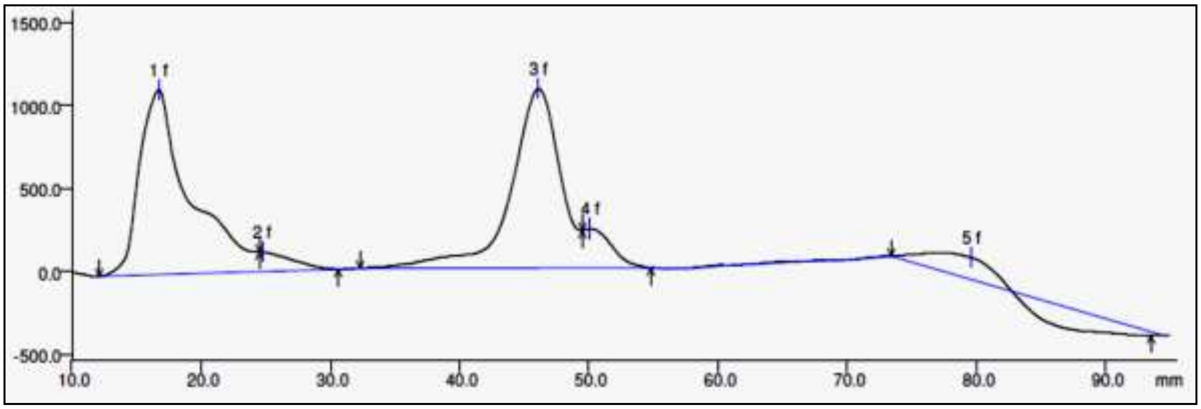

Figure 6 Chromatogram of aqueous extract of Amalaki (*Emblica officinalis)*

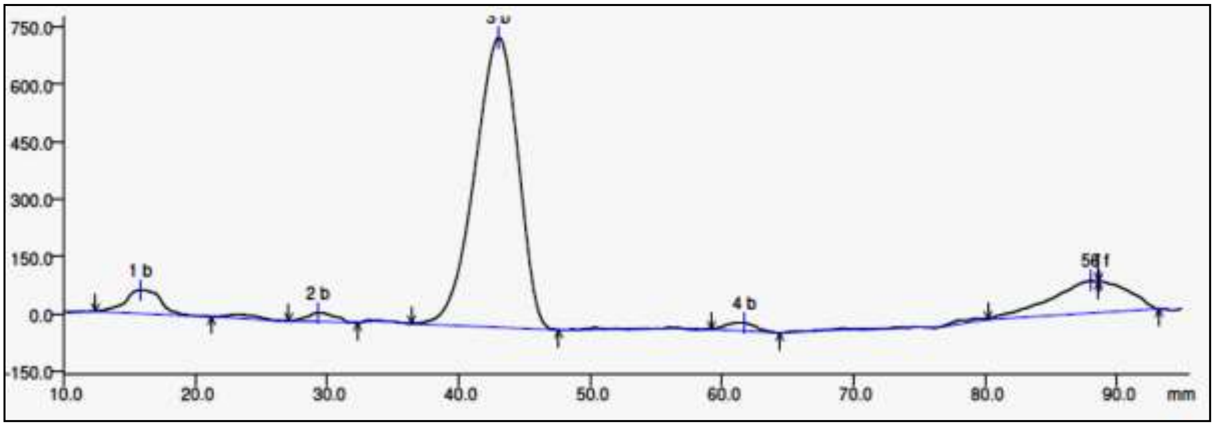

Figure 7 Chromatogram of alcoholic extract of Haritaki (*Terminalia chebula)*

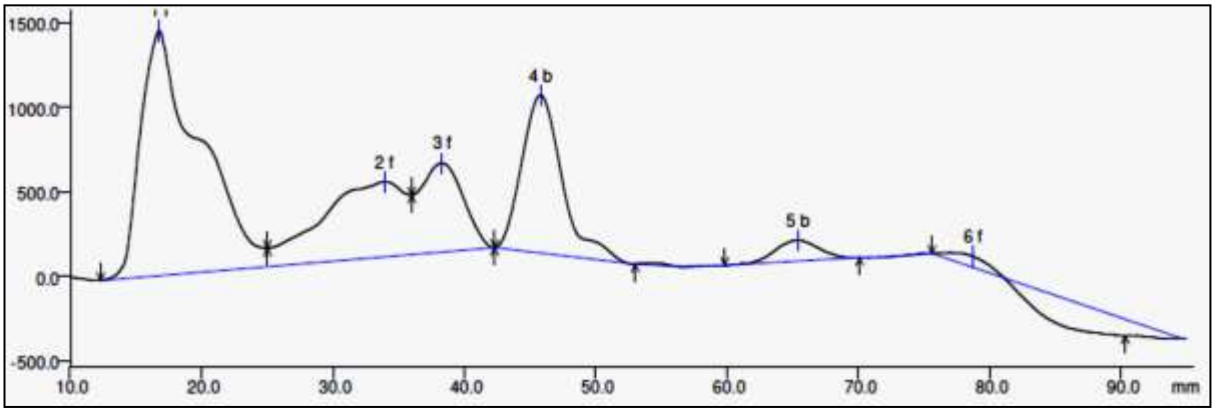

Figure 8 Chromatogram of alcoholic extract of Bibhitaki (*Terminalia bellirica)*

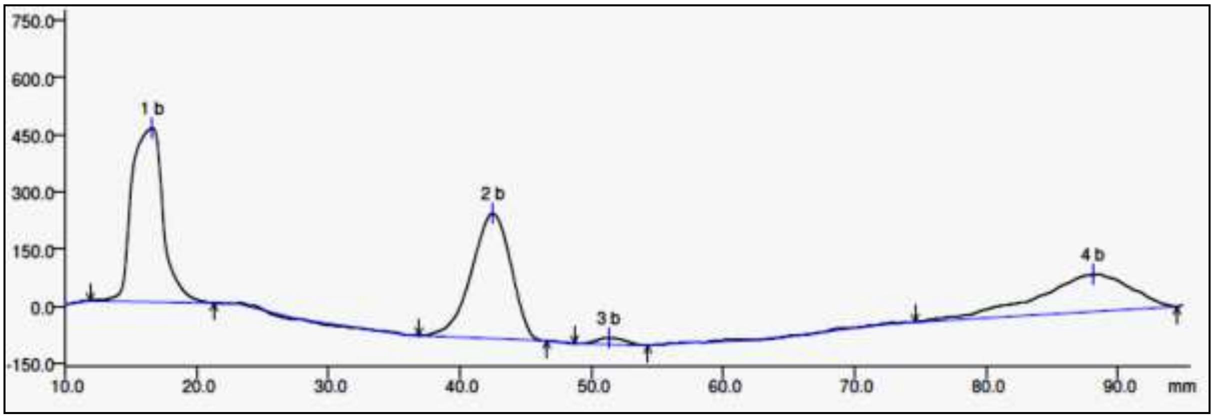

Figure 9 Chromatogram of alcoholic extract of *Amalaki (Emblica officinalis)*

Estimation of ellagic acid by HPTLC

Estimation of ellagic acid in different extracts was performed using HPTLC. The aqueous extract of Haritaki *(Terminalia chebula)* was found to contain 1.084 ± 0.08 %w/w, and alcoholic extract 0.250±0.07%w/w of ellagic acid. Aqueous extract of Bibhitaki *(Terminalia bellirica)* was found to contain 0.329±0.08%w/w, while alcoholic extract showed 1.163±0.02%w/w of ellagic acid.

Aqueous extract of Amalaki *(Emblica officinalis)* showed 1.282±0.05%w/w, while alcoholic extract showed 0.259±0.02%w/w of ellagic acid.

Table 2 Determination of ellagic acid by HPTLC

**Name of Extract**	Ellagic acid (%w/w)
Aqueous extract of Haritaki *(Terminalia chebula)*	1.084 ± 0.08
Aqueous extract of Bibhitaki *(Terminalia bellirica)*	0.329±0.08
Aqueous extract of Amalaki *(Emblica officinalis)*	1.282±0.05
Alcoholic extract of Haritaki *(Terminalia chebula)*	0.250±0.07
Alcoholic extract of Bibhitaki *(Terminalia bellirica)*	1.163±0.02
Alcoholic extract of Amalaki *(Emblica officinalis)*	0.259±0.02

Values were expressed as Mean ± SEM (n=3)

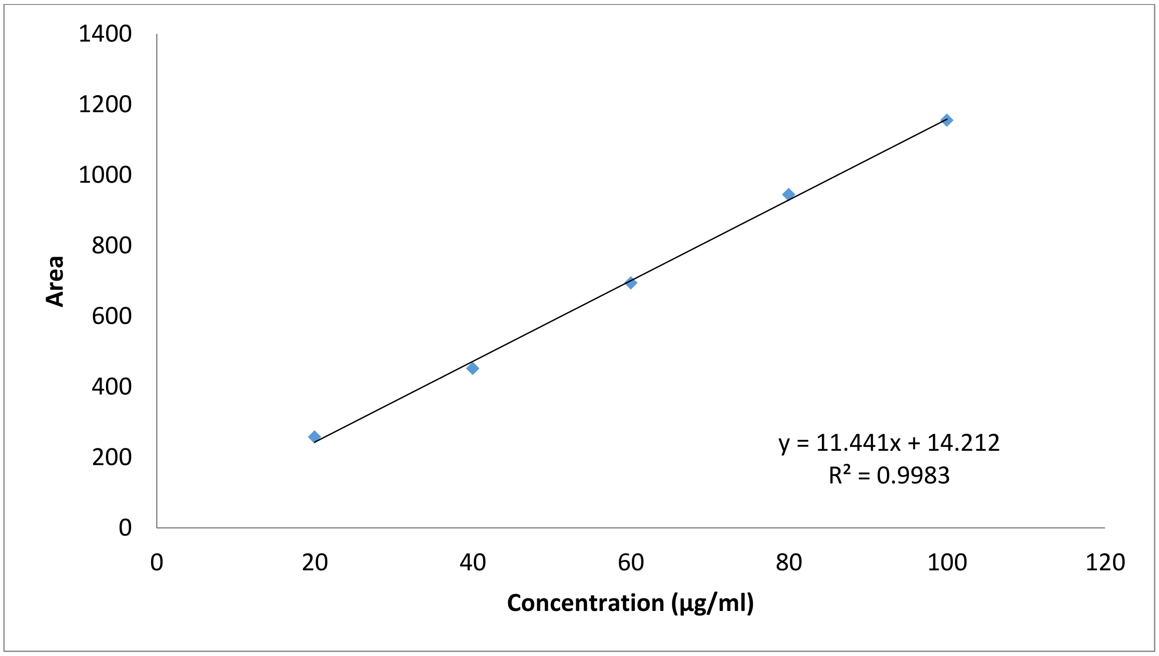

Figure 10 Calibration curve of ellagic acid by HPTLC

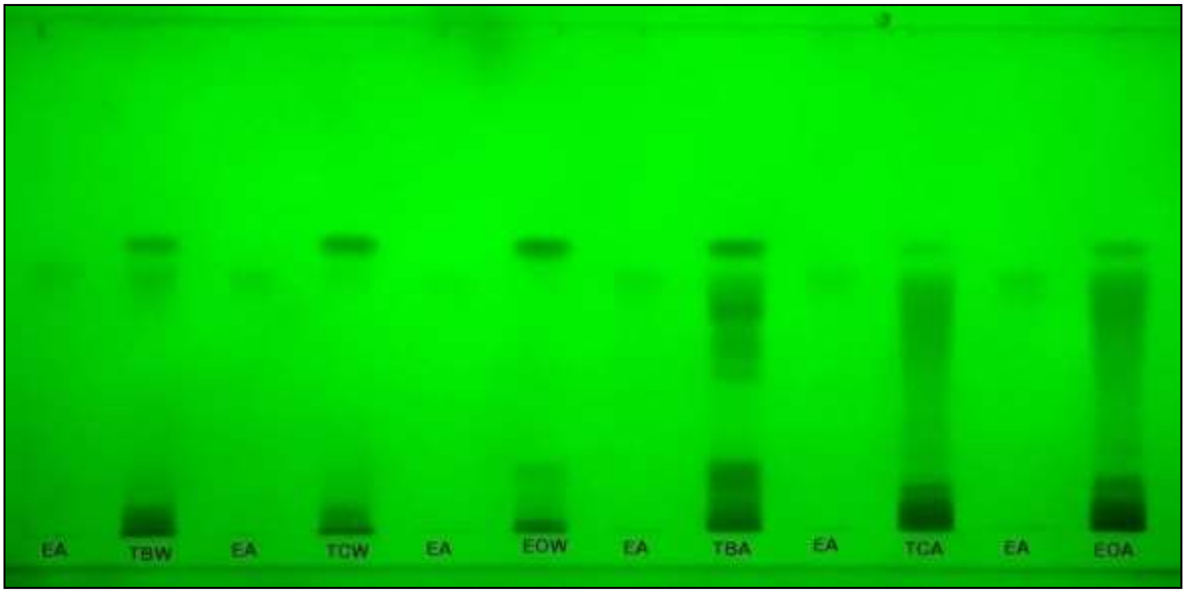

Figure 11 HPTLC of ellagic acid

EA: Ellagic acid, **TBW**: Aqueous extract of *Terminalia bellirica*, **TCW**: Aqueous extract of *Terminalia chebula*, **EOW**: Aqueous extract of *Emblica officinalis*, **TBA**: Alcoholic extract of *Terminalia bellirica*, **TCA**: Alcoholic extract of *Terminalia chebula*, **EOA**: Alcoholic extract of *Emblica officinalis*,

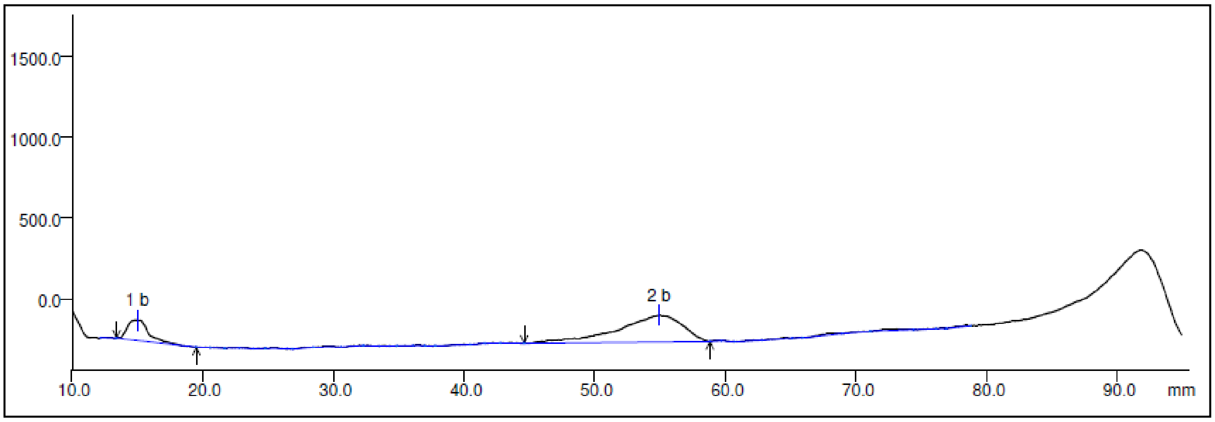

Figure 12 Chromatogram of standard ellagic acid

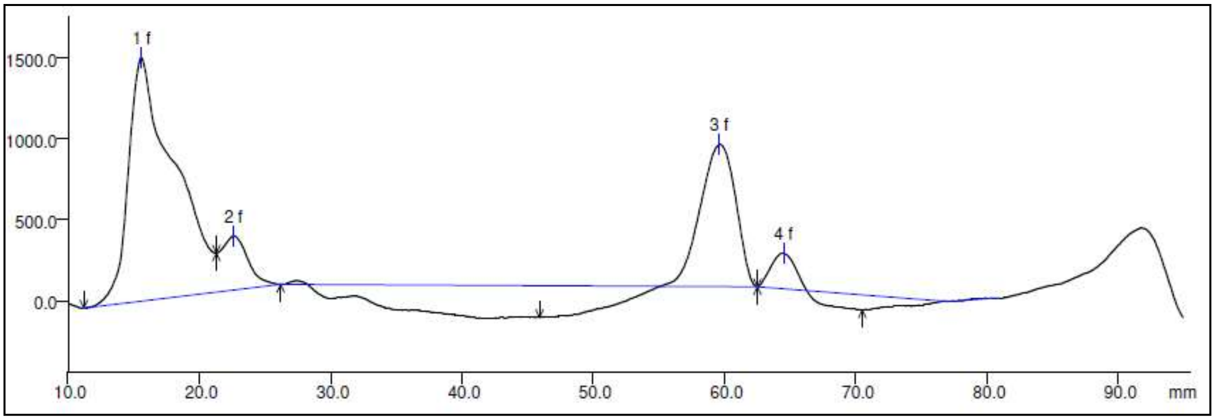

Figure 13 Chromatogram of aqueous extract of Haritaki (*Termianalia chebula)*

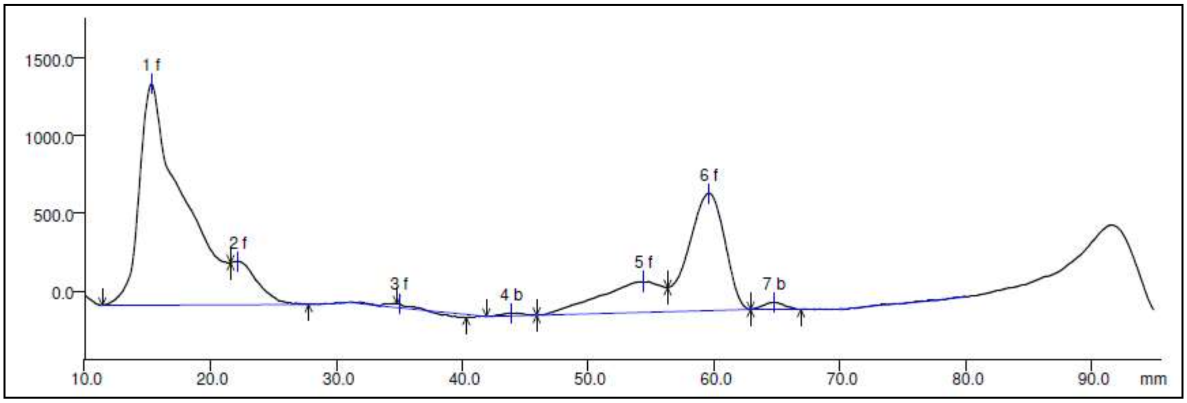

Figure 14 Chromatogram of aqueous extract of Bibhitaki (*Termianalia bellirica)*

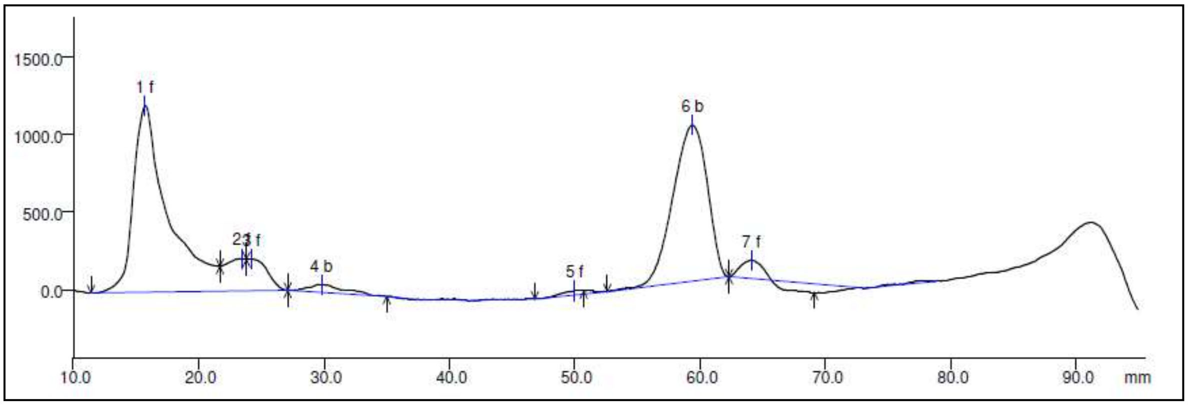

Figure 15 Chromatogram of aqueous extract of Amalaki (*Emblica officinalis)*

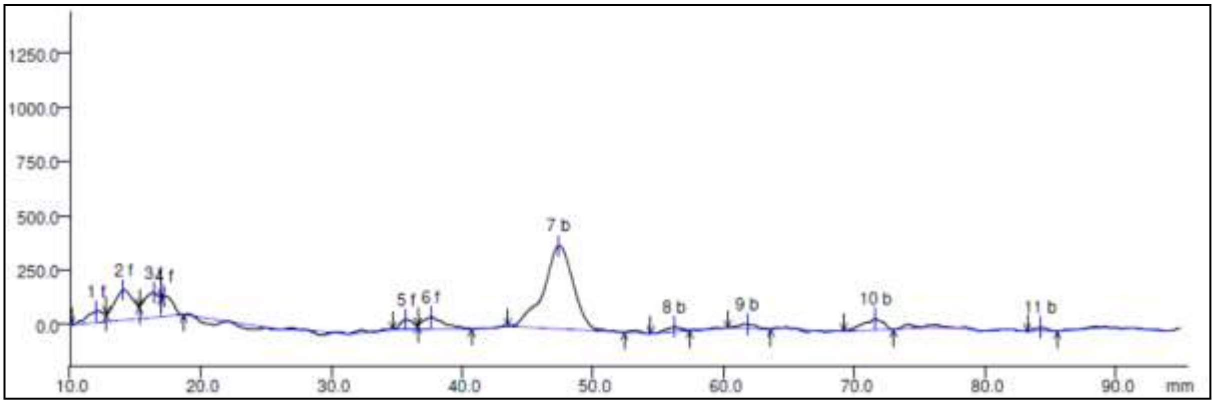

Figure 16 Chromatogram of alcoholic extract of Haritaki (*Terminalia chebula)*

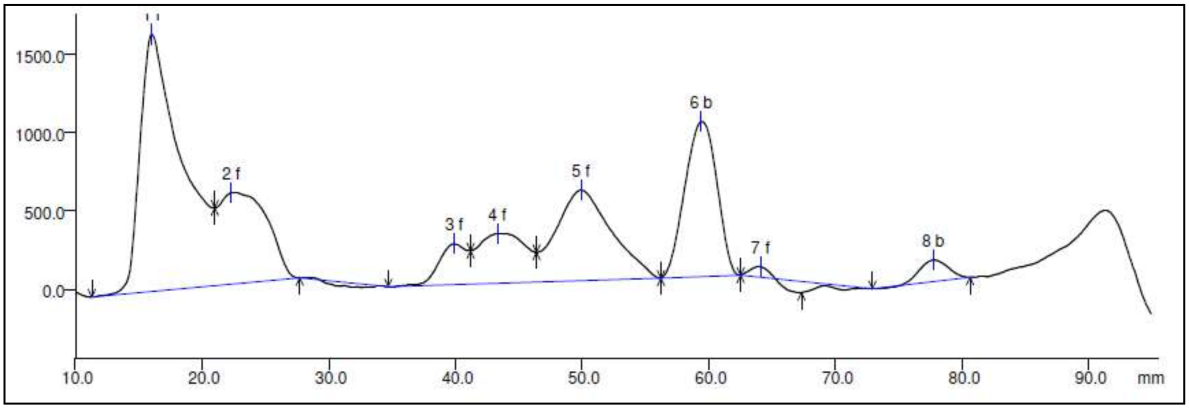

Figure 17 Chromatogram of alcoholic extract of Bibhitaki (*Terminalia bellirica)*

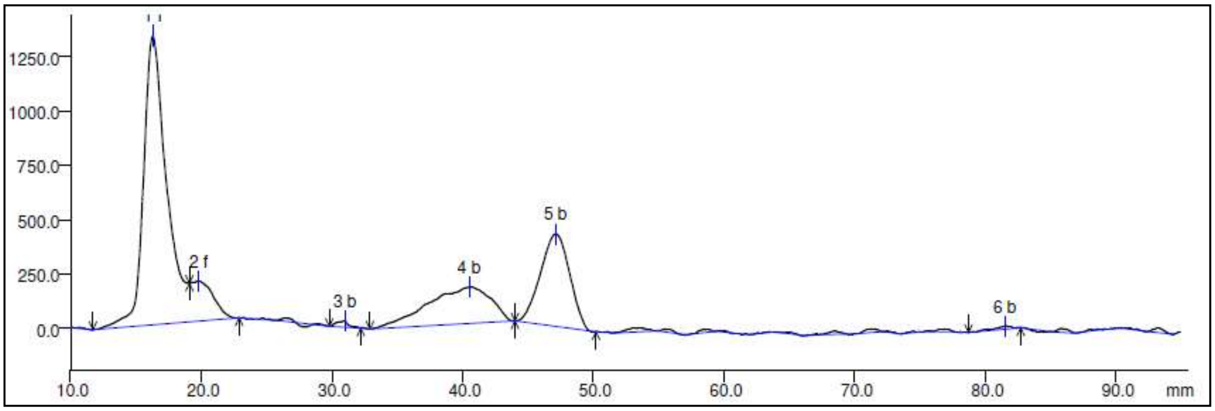

Figure 18 Chromatogram of alcoholic extract of Amalaki (*Emblica officinalis)*
